# Morphological characterization of intra-articular HMGB1 expression during the course of collagen-induced arthritis

**DOI:** 10.1186/ar2155

**Published:** 2007-03-30

**Authors:** Karin Palmblad, Erik Sundberg, Margarita Diez, Riikka Söderling, Ann-Charlotte Aveberger, Ulf Andersson, Helena Erlandsson Harris

**Affiliations:** 1 Department of Woman and Child Health, Karolinska Institutet, Astrid Lindgren Children's Hospital, SE-171 76 Stockholm, Sweden; 2 Department of Medicine, Rheumatology Research Unit, Center of Molecular Medicine L8:04,, Karolinska Institutet, SE-171 76 Stockholm, Sweden; 3 Department of Clinical Neuroscience, Neuroimmunology Unit, Center of Molecular Medicine L8:04, Karolinska Institutet, SE-171 76 Stockholm, Sweden

## Abstract

High-mobility group chromosomal box protein 1 (HMGB1) is a structural nuclear protein that promotes inflammation when present extracellularly. Aberrant, extracellular HMGB1 expression has been demonstrated in human and experimental synovitis. The aim of the present study was to elucidate the temporal and spatial expression of HMGB1 compared to that of the central mediators tumor necrosis factor (TNF) and interleukin-1-beta (IL-1β) during the course of collagen-induced arthritis. Thus, Dark Agouti rats were immunized with homologous type II collagen and synovial tissue specimens were obtained at various time points prior to and during the course of clinical arthritis. Local cytokine responses were assessed by immunohistochemistry and by *in situ *hybridization. We demonstrate a distinct nuclear expression of HMGB1 at early disease-preceding time points. Preceding clinical onset by a few days, cytoplasmic HMGB1 expression was evident in synoviocytes within the non-proliferative lining layer. Pronounced cytoplasmic and additional extracellular HMGB1 expression coincided with the progression of clinical disease. In advanced arthritis, the number of cells with cytoplasmic HMGB1 expression was quantitatively comparable to that of cells expressing TNF and IL-1β. Interestingly, although HMGB1 was abundantly expressed throughout the inflamed synovium at a protein level, upregulation of HMGB1 mRNA was restricted mainly to areas of cartilage and bone destruction. In conclusion, these new findings implicate a role for HMGB1 in both inducing and perpetuating inflammatory events of significant importance in the destructive processes in chronic arthritis.

## Introduction

Rheumatoid arthritis (RA) is characterized by chronic inflammation of multiple joints which leads to the marked destruction of cartilage and bone. Although the etiology of RA is still unknown, evidence is accumulating that, once initiated, the inflammatory process in the synovial tissue is dominated by activated monocytes/macrophages and fibroblasts. Cytokines derived from these cell types are abundantly expressed, and it is now commonly accepted that tumor necrosis factor (TNF) and interleukin-1 (IL-1) are pivotal mediators in the pathogenesis of RA, providing validated targets for successful therapy [1,2].

High-mobility group chromosomal box protein 1 (HMGB1), previously called HMG-1 or amphoterin, is an abundant nuclear component in all eukaryons [3]. Although widely studied as a DNA-binding protein, HMGB1 has recently been shown to possess important extracellular functions as well. Outside the cell, HMGB1 plays a critical role as a pro-inflammatory cytokine that mediates lipopolysaccharide (LPS) lethality, acute lung injury, and smooth muscle cell migration and induces the release of TNF and IL-1 from macrophages and dendritic cells [4-7]. In both experimental septic shock and acute lung injury, treatment targeting HMGB1 ameliorated inflammation and improved survival. HMGB1 translocation to the extracellular milieu can occur via two separate mechanisms. Through a regulated process, stimulated inflammatory cells may actively secrete HMGB1 [8-11]. In addition, HMGB1 can be passively released during disintegration of necrotic cells. In apoptotic cells, which do not trigger inflammation, HMGB1 is tightly bound to the chromatin, preventing extracellular release. Necrotic *Hmgb1*^-/- ^cells mediate a minimal inflammatory response, thus implying that HMGB1 is a critical factor connecting unprogrammed/necrotic cell death to inflammation [12].

Recent evidence implicates a role for HMGB1 in the pathogenesis of arthritis (reviewed in [13]). We and others have demonstrated local overexpression of cytoplasmic and extracellular HMGB1 in synovial biopsy specimens in RA and experimental arthritis [14,15]. Intra-articular injection of HMGB1 in mice induces arthritis, and treatment with HMGB1 antagonist attenuates collagen-induced arthritis (CIA) in rats and mice [16,17].

CIA is a widely used animal model that mimics the joint inflammation evident in human RA. The Dark Agouti rat is particularly susceptible to CIA, presenting an erosive chronic relapsing disease in 100% of immunized animals when induced with homologous collagen type II emulsified with Freund's incomplete adjuvant [18]. This model was used in the present study to elucidate characteristics of HMGB1 expression in comparison with the well-characterized cytokines TNF and IL-1β in the initiation and progression of arthritis. Local cytokine responses were determined at the protein level by means of recently developed immunohistochemical techniques that enable discrimination of the localization of HMGB1 in cellular compartments. In addition, HMGB1 mRNA expression was determined using *in situ *hybridization techniques.

## Materials and methods

### Induction and evaluation of experimental arthritis

Male Dark Agouti rats weighing 220 to 230 g were bred and kept at the animal unit at Karolinska Hospital in Stockholm, Sweden. The light/darkness cycle was 12 hours, and the rats were fed standard rodent chow and water *ad libitum*. The health status of the animals was monitored according to guidelines of the Swedish Veterinary Board (SVA), and the animals were reported to be free of screened pathogens. The Stockholm North Ethical Committee, Sweden, approved all of the procedures during the experiments. On day 0, 28 rats were immunized intradermally in the base of the tail with rat type II collagen emulsified with Freund's incomplete adjuvant (Difco, Detroit, MI, USA) as previously described [18]. With this protocol, chronic polyarthritis is expected to develop in 100% of the animals and clinical onset occurs at approximately day 15 after immunization. The paws of the rats were monitored daily for visual inflammatory signs such as erythema and swelling by means of a previously described scoring system [19]. Arthritis was graded semiquantitatively on a scale of 0 to 4 for each paw. An arthritis index that expressed a cumulative score for all paws (maximum possible value = 16) was calculated for each animal.

### Preparation of samples for immunohistochemical analysis

Thirty-two animals were included in this longitudinal trial. Four unimmunized animals were sacrificed at day 0 as normal controls. Three early time points (3, 6, and 10 days post-immunization [p.i.]), the time point of expected onset (day 15 p.i.), the time point for expected maximal clinical severity of arthritis (day 21 p.i.), the time point for transition to a chronic phase of disease (day 28 p.i.), and a late time point (day 38 p.i.) were selected, and four animals per time point were sacrificed. To examine and compare local histology, rats were perfused *in vivo *with paraformaldehyde solution; paws were then dissected and decalcified using a modification of a protocol previously described [20]. Briefly, animals were deeply anesthetized by intraperitoneal injection of a mixture of equal volumes of Hypnorm^® ^(fentanyl citrate 0.315 mg/ml and fluanisone 10 mg/ml; Janssen Pharmaceutica N.V., Beerse, Belgium) and Dormicum^® ^(midazolam 1 mg/ml; Roche, Stockholm, Sweden), diluted 1:2 in sterile water, in which 800 μl per 200 g of animal's body weight was given. Central intra-arterial perfusion with phosphate-buffered saline (PBS) preceded perfusion with the fixative, which consisted of 4% (wt/vol) paraformaldehyde (Merck, Darmstadt, Germany) in 0.2 M Sörensen phosphate buffer, pH 7.2, containing 0.2% picric acid (Riedel-de Haën, Seelze, Germany). Ankle joints were dissected and immersed in the same fixative overnight at room temperature and thereafter thoroughly washed in PBS twice daily for three to four days until clear of picric acid. The joint specimens were then subjected to demineralization in a 4% (wt/vol) EDTA (ethylenediaminetetraacetic acid) (Sigma-Aldrich, St. Louis, MO, USA) solution containing 0.2 M sodium cacodylate (Sigma-Aldrich), pH 7.3, for approximately four weeks, followed by eight days in 20% (wt/vol) sucrose (Sigma-Aldrich) in 0.1 M Sörensen phosphate buffer, pH 7.2, containing 0.01% (wt/vol) sodium azide (Sigma-Aldrich). The ankle joints were then cut in saggital sections of 7 to 8 μm in thickness by means of a Leica Cryostat (Leica, Wetzlar, Germany). The sections were mounted directly on Superfrost slides (Novakemi AB, Stockholm, Sweden), air-dried at room temperature, and subsequently stored at -70°C until used for staining. Because the arthritic lesions were symmetrical and scoring in the hind paws was equal, only one paw per rat was studied.

### Immunohistochemical stainings

To detect expressions of HMGB1, TNF, and IL-1β, sections were stained according to immunohistochemical methods previously described by us [21]. The primary antibodies used were a peptide affinity-purified polyclonal rabbit anti-HMGB1 antibody (cat. no. 556528; BD Pharmingen, San Diego, CA, USA), a polyclonal ligand affinity-purified rabbit anti-rat TNF (8–14; U-CyTech biosciences, Utrecht University, Utrecht, The Netherlands), and a polyclonal ligand affinity-purified goat anti-rat IL-1β (AF-501-NA; R&D Systems, Inc., Minneapolis, MN, USA). The HMGB1 antibody was used at a final concentration of 1 μg/ml, and the TNF and IL-1β antibodies were used at a final concentration of 2 μg/ml.

In each assay, controls for specificity of cytokine stainings based on parallel staining studies omitting the primary antibody or using primary isotype-matched immunoglobulin (Ig) of irrelevant antigen specificity at the same concentration as the cytokine-detecting antibodies were included. The irrelevant control antibodies used in the present study were fractioned rabbit Ig (no. XO936; DakoCytomation, Glostrup, Denmark) and goat anti-human IL-2 (AF-202; R&D Systems, Inc.). The specificities of extracellular and intracellular cytokine immunoreactivities were verified by their complete inhibition in blocking experiments with preabsorption of the cytokine-specific antibody with recombinant target cytokine prior to staining. In addition, a morphology of HMGB1 expression similar to the stainings demonstrated in this report using the BD Pharmingen anti-HMGB1 antibody was obtained using a polyclonal peptide affinity-purified rabbit anti-HMGB1 antibody purchased from Innovagen AB (Lund, Sweden). These two antibody preparations recognize separate epitopes of the HMGB1 molecule.

### Evaluation of the stained sections

By means of a Polyvar II microscope (Reichert-Jung, now part of Leica Microsystems Nussloch GmbH, Nussloch, Germany) connected to a 3-CCD (charge-coupled device) color camera (DXC-750P; Sony Corporation, Tokyo, Japan), slides were evaluated by two independent observers blinded to the identity of the specimens. All animals were studied in at least three separate staining experiments for each given cytokine. The relative frequencies of positively stained cells in the articular tissue were estimated and assigned an expression score on a scale of 0 to 4: 0, negative cells; 0.5, less than 1%; 1, 1% to 5%; 2, 5% to 20%; 3, 20% to 50%; and 4, more than 50% positively stained cells.

### Immunofluorescence two-color staining

To determine the phenotype of the HMGB1-releasing cells, we performed a two-color staining of HMGB1 and ED1 (Serotec Ltd, Oxford, UK), a surface membrane antigen expressed on rat macrophages, monocytes, and dendritic cells, by means of a modified staining protocol. Briefly, PBS supplemented with 0.1% (wt/vol) saponin (Riedel-de Haën) was used in all subsequent washes and incubation steps. Endogenous biotin was blocked with avidin for 30 minutes and with biotin for an additional 15 minutes (avidin/biotin blocking kit; Vector Laboratories, Burlingame, CA, USA), both substituted with 0.1% saponin. Sections were then incubated overnight with a mixture of primary antibodies directed against HMGB1 and ED1, supplemented with 0.1% Aurion BSA-c (acetylated bovine serum albumin) (10%) (Scandinavian Medical Services, Helsingborg, Sweden) to reduce background staining due to non-specific binding sites. HMGB1 staining was developed with a secondary biotin-labeled Fab_2_-fragmented donkey anti-rabbit antibody (Jackson ImmunoResearch Laboratories, Inc., West Grove, PA, USA) diluted 1:1,000, followed by the streptavidin-conjugated fluorophore Oregon green at 2 μg/ml; both incubations were performed for 30 minutes. Subsequently, after another blocking with avidin for 30 minutes and biotin for an additional 15 minutes, the surface antigen ED1 staining was developed with biotin-labeled Fab_2_-fragmented donkey anti-mouse antibody (Jackson ImmunoResearch Laboratories, Inc.) diluted 1:1,000 for 30 minutes, followed by a 30-minute incubation with streptavidin-conjugated Alexa 546 (red fluorophore) coupled to avidin diluted 1:400 in PBS-saponin. Slides were air-dried and then mounted with PBS-buffered glycerol. Slides were examined with a Polyvar 2 UV microscope (Leica Microsystems Nussloch GmbH) equipped with a 200-W mercury lamp.

### *In situ *hybridization

A 50-base pair oligonucleotide probe for HMGB1 (TCTTCTTCCTCCTCTTCCTCATCCTCTTCATCCTCCTCGTCGTCTTCCTC) and a random probe having no similarities to known sequences (GenBank, National Institutes of Health, Bethesda, MD, USA) were synthesized (DNA Technology A/S, Århus, Denmark). *In situ *hybridization was performed as previously described [22]. Briefly, oligonucleotide probes were labeled with ^33^P-dATP (DuPont-New England Nuclear, now part of PerkinElmer Life and Analytical Sciences, Inc., Waltham, MA, USA) at the 3' end by means of terminal deoxynucleotidyltransferase (Amersham, now part of GE Healthcare, Little Chalfont, Buckinghamshire, UK) and purified through QIAquick spin columns (Qiagen GmbH, Hilden, Germany). Sections were hybridized overnight at 42°C in humidified boxes with 0.5 ng of labeled probe (1 to 4 × 10^6 ^cpm/l) per slide in a hybridization cocktail and rinsed 5 × 15 minutes in saline sodium citrate at 60°C. As a control, an excess (×100) of cold probe was added to the hybridization cocktail. Tissue sections were dehydrated, air-dried, dipped in NTB2 nuclear track photographic emulsion (Eastman Kodak, Rochester, NY, USA), and exposed for 7 to 14 days at 8°C. Dipped slides were developed for 4 minutes in D19 (Eastman Kodak), fixed in Unifix (Eastman Kodak) for 7 minutes, and rinsed in tap water for 20 minutes. After air-drying, sections were counterstained with eosin-hematoxylin and mounted.

## Results

### Cytokine expression before onset of arthritis

Immunohistochemical stainings were performed to study the spatial and temporal cytoplasmic expression of the novel cytokine HMGB1 compared to those of IL-1β and TNF in synovial tissue specimens at different time points after immunization with type II collagen. In synovial sections from animals sacrificed before the onset of disease (before day 15 p.i.), the synovial tissue appeared (as expected) non-proliferative, containing only a few cell layers.

**Figure 1:**
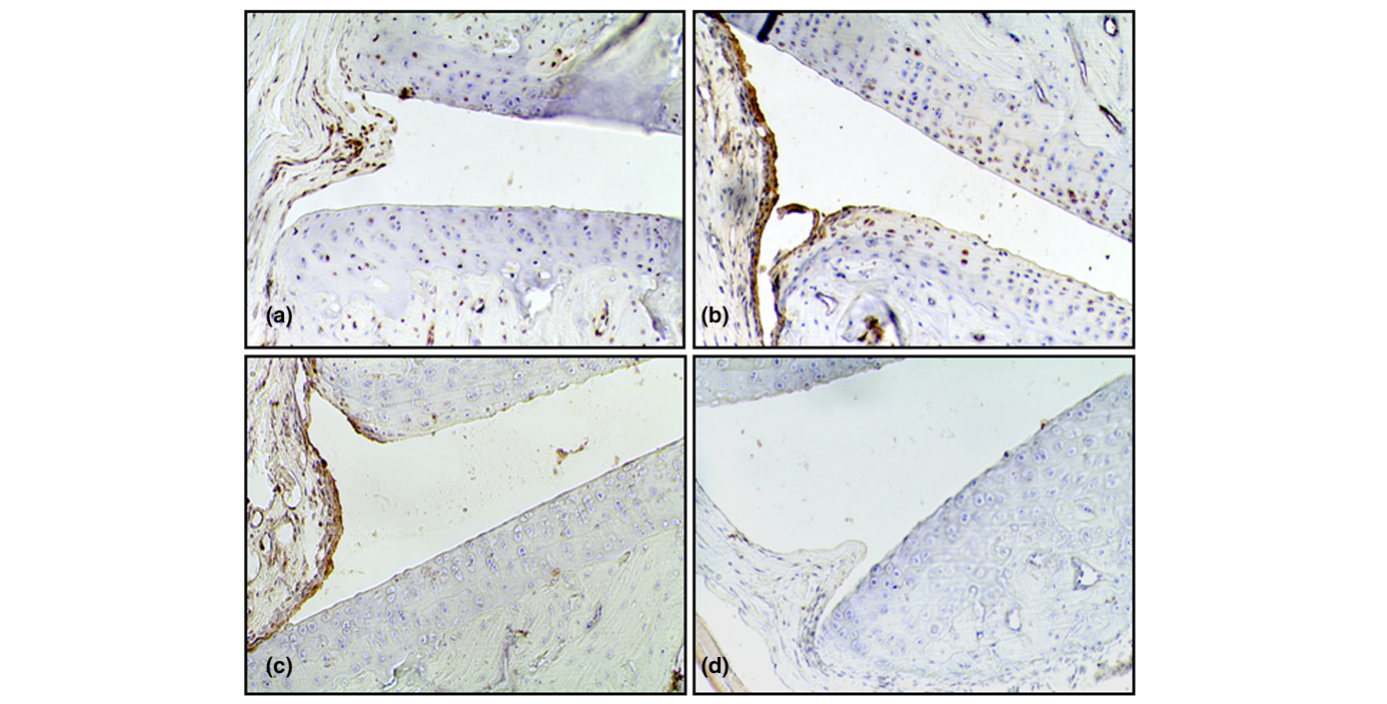
Synovial cytokine expression at an early disease-preceding time point. Representative micrographs illustrating immunohistochemical staining of cryocut synovial tissue for expressions of high-mobility group chromosomal box protein 1 (HMGB1) **(a)**, interleukin-1-beta (IL-1β) **(b)**, and tumor necrosis factor (TNF) **(c) **three days after immunization. A thin, non-proliferative synovia is evident at this disease-preceding time point. TNF- and IL-1β-expressing cells are located in superficial parts of the synovial lining layer. HMGB1 expression was restricted to cell nuclei at this early time point. **(d) **A representative section is stained with irrelevant control antibody. Original magnification ×125.

**Figure 2:**
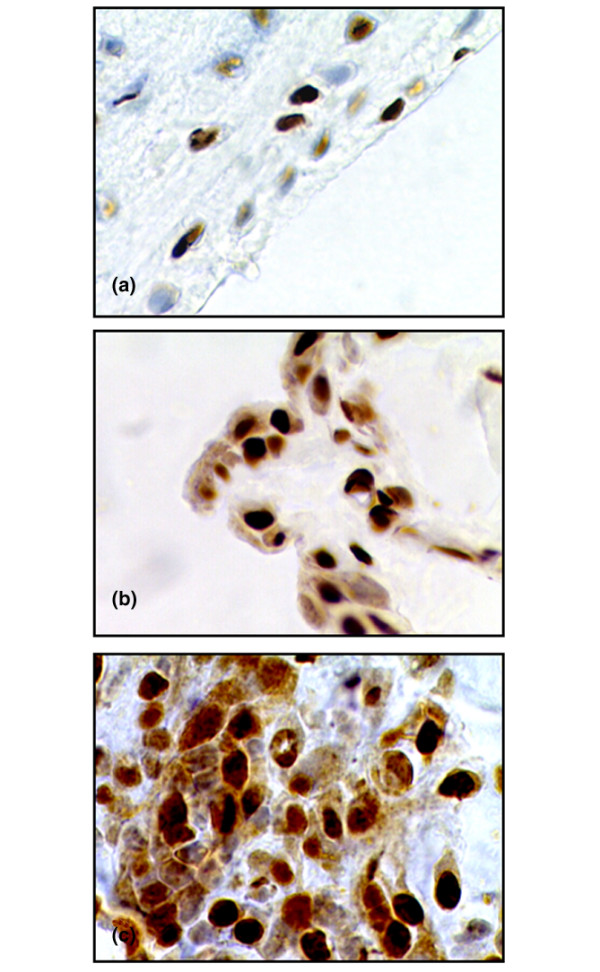
High-mobility group chromosomal box protein 1 (HMGB1) expression at different time points after immunization. Representative micrographs illustrating immunohistochemical staining of HMGB1. **(a) **In the non-proliferative synovial membrane of an unimmunized animal, a nuclear HMGB1 deposition is evident. **(b) **In addition to the nuclear expression, cytoplasmic HMGB1 staining appears in a large portion of cells in the synovial membrane 10 days after immunization, a time point preceding the expected clinical disease onset by 5 days. **(c) **An arthritic lesion, 28 days after immunization, in which an additional extracellular presence of HMGB1 is indicated by a brownish extracellular immunoreactivity surrounding cells displaying cytoplasmic HMGB1 staining. Original magnification ×500.

Joint tissue specimens at these disease-preceding time points revealed a strict nuclear cellular localization of detectable HMGB1 in virtually all cells in the synovial membrane (Figures 1a and 2a). Although cytoplasmic HMGB1 expression was scarce at early disease-preceding time points, more evident signs of extranuclear deposition of HMGB1 appeared at day 10 p.i. (Figure 2b), thus preceding the time for clinical disease onset by five days. This extranuclear HMGB1 appeared as a general cytoplasmic staining in a large portion of cells in the lining layer. At this time point, the synovial membrane remained unproliferative with an appearance indistinguishable from unimmunized animals.

**Figure 3:**
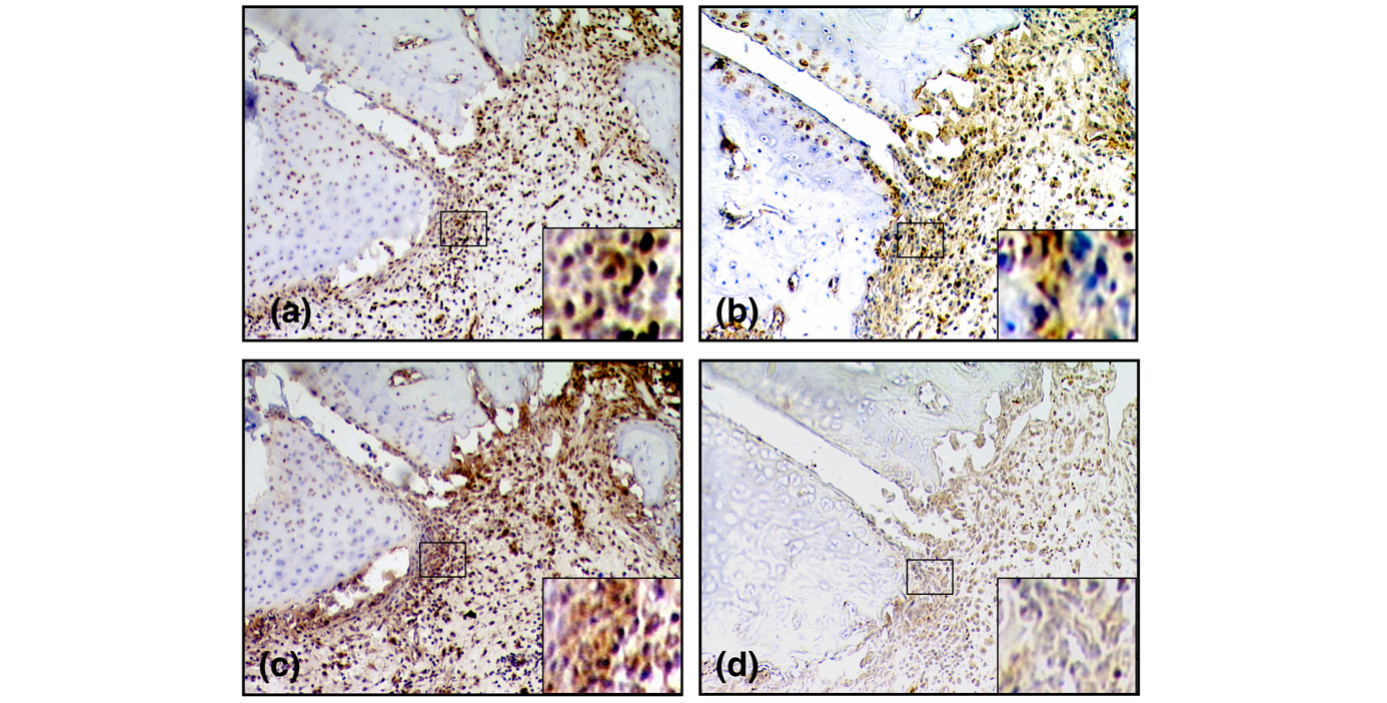
The number of cells expressing extranuclear high-mobility group chromosomal box protein 1 (HMGB1) is quantitatively comparable to the number of cells expressing tumor necrosis factor (TNF) and interleukin-1-beta (IL-1β) in arthritic joints. Representative micrographs illustrating immunohistochemical staining of synovial tissue from an arthritic animal at day 21 after immunization. Sequential cryocut sections were analyzed for expressions of HMGB1 **(a)**, IL-1β **(b)**, and TNF **(c)**. Abundant expressions were demonstrated for all three cytokines. **(d) **A section stained with an irrelevant isotype-matched control. Original magnification ×125.

Scattered TNF- and IL-1β-expressing cells could even be recognized in the superficial cell layer of the synovial lining in unimmunized animals (Figure 1b,c). Sections from days 3 to 15 p.i. displayed a rather congruent picture. One difference was evident between expressions of these two cytokines. Chondrocytes expressing IL-1β could be detected at all time points with a more prominent expression in the superficial articular cartilage layer, whereas the cartilage remained negative for TNF expression throughout the study (Figures 1 and 3; Table 1). The chondrocyte staining pattern of IL-1β resembled that of HMGB1 (Figures 1a,b and 3a,b).

**Table 1:** Expression of extranuclear HMGB1 compared to those of TNF and IL-1β

		Lining			Sublining			Destructive zone			Cartilage			Vessels		
Days p.i.	M.A.I.	HMGB1	TNF	IL-1β	HMGB1	TNF	IL-1β	HMGB1	TNF	IL-1β	HMGB1	TNF	IL-1β	HMGB1	TNF	IL-1β

		1			1			1			1			1		
0	0	0	0.5	0.5	0	0	0	-	-	-	1	0	1	0	0	0
3	0	0	0.5	0.5	0	0	0	-	-	-	1	0	1	0	0	0
6	0	0	0.5	0.5	0	0	0	-	-	-	1	0	1	0	0	0
10	0	0.5	0.5	0.5	0.5	0	0	-	-	-	1	0	1	0	0	0
15	3.2 ± 1.8	1	1	1	1	0.5	0	-	-	-	1	0	1	0	0.5	0
21	9.8 ± 3.3	2	4	2	3	3	3	3	3	3	2	0	2	1	3	1
28	11.0 ± 2.3	2	4	2	3	3	3	3	3	3	2	0	2	1	3	1
38	11.8 ± 2.6	2	4	2	3	3	3	3	3	3	2	0	2	1	3	1

The relative frequencies of positively stained cells in the articular tissue were estimated by immunohistochemistry and assigned an expression score on a scale of 0 to 4: 0, negative cells; 0.5, less than 0.5%; 1, 0.5% to 5%; 2, 5% to 20%; 3, 20% to 50%; and 4, more than 50% positively stained cells. For clinical evaluation, a mean arthritis index (M.A.I.) was calculated for the group of four animals studied per time point and expressed as the mean ± standard deviation. The destructive zone did not appear until day 21 p.i. and was defined as synovial tissue adjacent to areas of cartilage and bone destruction. HMGB1, high-mobility group chromosomal box protein 1; IL-1β, interleukin-1-beta; p.i., post-immunization; TNF, tumor necrosis factor.

### Cytokine expression after onset of arthritis

At the time point of clinical disease onset (day 15 p.i.), the first signs of cell infiltration were noted and the synovial membrane increased in thickness. Although the arthritis index varied within the group of four animals studied per time point, the estimated expression scores of the cytokine expression appeared to be very similar within the group with reproducible results in at least three staining experiments. A substantial number of the first infiltrating inflammatory cells expressed HMGB1 in their cytoplasm, which was apparently more pronounced than the expressions of TNF or IL-1β.

**Figure 4:**
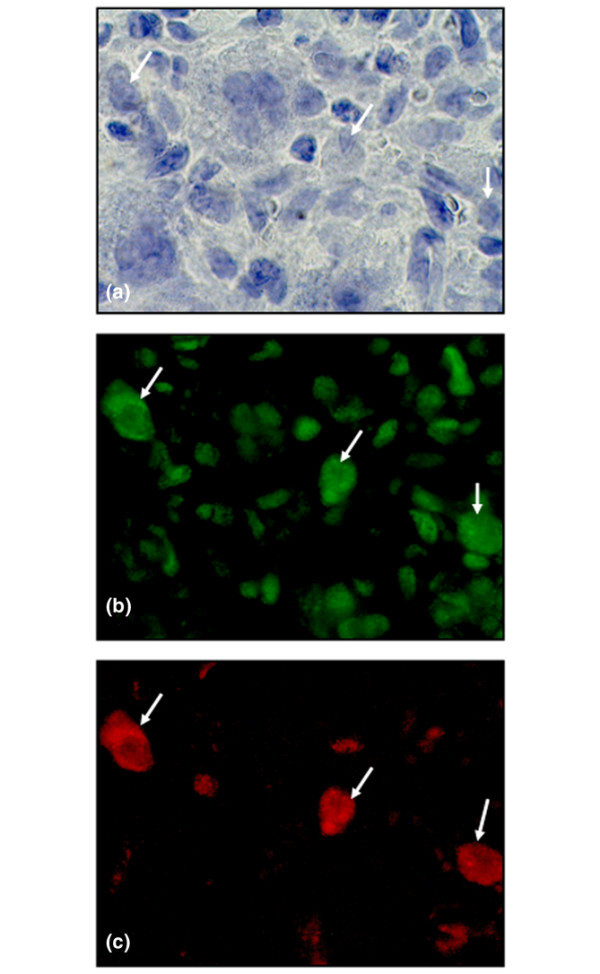
A substantial portion of cells expressing cytoplasmic high-mobility group chromosomal box protein 1 (HMGB1) are also ED1-positive. **(a) **Micrograph illustrating a high magnification of inflamed synovial tissue stained with hematoxylin (arrows). **(b) **The intranuclear HMGB1 staining (Oregon green) of resident cells is evident. A substantial portion of cells express extranuclear HMGB1 (arrows). **(c) **A substantial portion of cells expressing extranuclear HMGB1 were also ED1-positive (Red Alexa 546) (arrows). ED1 is a surface membrane antigen expressed on rat macrophages, monocytes, and dendritic cells. Original magnification ×800.

However, a more evident presence of all three studied cytokines coincided with the progression of clinical disease (Table 1). Accordingly, maximal cytokine expression was recorded from day 21 p.i. onward, corresponding to maximal paw swelling, cell infiltration, and manifestation of erosive changes in cartilage and bone (Figure 3). At these time points, the number of TNF-expressing cells dominated quantitatively throughout the synovial tissue, the most abundant expression being within the lining layer. Both spatial and quantitative aspects of extranuclear HMGB1 expression were similar to those of IL-1β, in which most of the expression was evident within sublining and pannus regions (Table 1). The most prominent staining of cytoplasmic HMGB1 and of IL-1β was located in erosive parts of synovial tissue close to cartilage and bone undergoing destruction. As opposed to expression of TNF, those of both cytoplasmic HMGB1 and IL-1β were lower within the lining layer. Cytoplasmic HMGB1 could be demonstrated in many macrophage-like cells, and the nuclear HMGB1 staining in a subset of these cells was clearly reduced or absent. Two-color staining revealed that a substantial number of cells with cytoplasmic HMGB1 expression were also positive for ED1, a marker for rat macrophages and dendritic cells (Figure 4). An extracellular presence of HMGB1 was indicated by a brownish immunoreactivity that encompassed cells displaying cytoplasmic HMGB1 staining in the inflamed synovium (Figure 2a).

Scattered cells were stained for all studied cytokines and were distributed in the interstitial tissue, perivascularly, and within the vessel endothelium. A quantitative difference was also evident in that almost all vessel endothelium cells stained positively for TNF, whereas several vessels remained unstained for IL-1β and a nuclear staining pattern dominated for HMGB1, although endothelium cells with cytoplasmic HMGB1 could also be visualized (Table 1). The expressions of all three studied pro-inflammatory cytokines were still prominent at days 28 and 38 p.i., when (in clinical terms) a transition of the acute inflammation to a chronic phase occurred.

### HMGB1 mRNA expression

**Figure 5:**
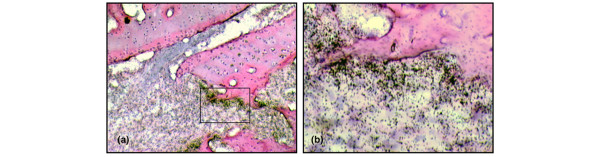
High-mobility group chromosomal box protein 1 (HMGB1) mRNA is predominantly upregulated in areas of tissue destruction. Micrographs illustrating intra-articular HMGB1 mRNA expression in advanced arthritis assessed by *in situ *hybridization. **(a) **A pronounced upregulation of HMGB1 mRNA is evident in synovial tissue close to cartilage and bone undergoing destruction, detailed in **(b)**.

A low mRNA expression was detected in most cells at all time points, even in paw sections of healthy unimmunized animals. Because HMGB1 is an abundantly displayed protein in all cell nuclei (where it binds to DNA-regulating transcription [23]), these findings were expected. The abundant extranuclear HMGB1 protein expression throughout the inflamed synovium in advanced arthritis, however, was not accompanied by an overall upregulation of HMGB1 mRNA. In contrast, upregulation of HMGB1 mRNA was restricted mainly to synovial tissue adjacent to areas with cartilage and bone destruction, where the expression was pronounced with cells expressing numerous grains (Figure 5; Table 2). No detectable labeling appeared after hybridization with cold probe or random probe.

**Table 2:** HMGB1 expression at a protein level compared to HMGB1 mRNA expression

		Lining		Sublining		Destructive zone		Cartilage		Vessels	
Days p.i.	M.A.I.	HMGB1 protein	HMGB1 mRNA	HMGB1 protein	HMGB1 mRNA	HMGB1 protein	HMGB1 mRNA	HMGB1 protein	HMGB1 mRNA	HMGB1 protein	HMGB1 mRNA

0	0	0	0.5	0	0.5	-	-	1	0.5	0	0.5
3	0	0	0.5	0	0.5	-	-	1	0.5	0	0.5
6	0	0	0.5	0	0.5	-	-	1	0.5	0	0.5
10	0	0.5	0.5	0.5	0.5	-	-	1	0.5	0	0.5
15	3.2 ± 1.8	1	0.5	1	0.5	-	-	1	0.5	0	0.5
21	9.8 ± 3.3	2	1	3	1	3	4	2	1	1	1
28	11.0 ± 2.3	2	1	3	1	3	4	2	1	1	1
38	11.8 ± 2.6	2	1	3	1	3	4	2	1	1	1

Most cells expressed a low HMGB1 mRNA labeling at all time points. To be able to compare the relative frequencies of cells with HMGB1 protein expression to those of cells with upregulated HMGB1 mRNA expression, an estimation of expression scores similar to that used in Table 1 was used for *in situ *results; cells with numerous grains were regarded as positive. HMGB1, high-mobility group chromosomal box protein 1; M.A.I., mean arthritis index; p.i., post-immunization.

## Discussion

In the present report, we provide evidence that, in rats with CIA, the number of synovial cells expressing cytoplasmic HMGB1 may be quantitatively comparable with the number of cells expressing the well-characterized cytokines TNF and IL-1β. HMGB1 is released as a late mediator during acute inflammation and plays a crucial role in the pathogenesis of systemic inflammation in sepsis after the early mediator response has resolved [4,24]. We thus anticipated analogous results in the present study of chronic inflammation, with synovial expression of TNF and IL-1β preceding that of HMGB1. However, we did not observe a distinct, sequential order of appearance of the three cytokines studied. At early disease-preceding time points, cellular HMGB1 expression was almost exclusively restricted to the nuclear compartment. Interestingly, a more evident extranuclear staining pattern for HMGB1 was noted in resident cells in the synovium even a few days before the onset of clinical disease. In addition, at the time point of arthritis onset, a substantial number of the infiltrating inflammatory cells expressed HMGB1 in their cytoplasm, apparently more than cells expressing TNF or IL-1β. Thus, in the context of chronic inflammation, HMGB1 may be considered an early mediator. This is in concordance with the demonstration of HMGB1 as an early mediator of inflammation following acute, local organ injury in liver ischemia reperfusion [25] as well as in post-ischemic brain injury [26].

All three macrophage-derived cytokines studied in this report were abundantly detected in synovial tissues with established arthritis. However, some clear differences were also discernible. A distinct TNF expression was observed in the lining layer as well as in sublining areas in synovitis, whereas HMGB1 and IL-1β expressions were most often restricted to the sublining areas. HMGB1 and IL-1β were abundantly displayed in chondrocytes, especially in those located superficially in the articular cartilage, whereas no TNF was detectable in chondrocytes at any time point. The discrepancy between TNF and IL-1β expressions in chondrocytes was unexpected. Although the destructive effects of IL-1 on cartilage and bone are well recognized, the biological implication that chondrocytes also express IL-1β remains unclear. The similarities between IL-1β and HMGB1 expressions in synovitis are noteworthy. Both cytokines lack a signal peptide [27,28], and it was recently shown that both are secreted by myeloid cells through a non-classical pathway involving regulated exocytosis of secretory lysosomes [8].

Rheumatoid synovium is characterized by excessive growth and invasion into adjacent tissues, including bone and cartilage. In many ways, it behaves and appears like a locally invasive tumor in the joints. Extracellular HMGB1 is known to bind to several components of the plasminogen activation system and to enhance the activity of tissue plasminogen activator [29] and matrix metalloproteinases 2 and 9 [30]. Degradation of extracellular matrix proteins is an important step in cell migration processes. The HMGB1-promoted increase of extracellular protease activity might enable cells to migrate and invade, analogous to the migratory response elicited in smooth muscle cells [31]. Because HMGB1 initiates endothelial growth as well as endothelial cell migration and sprouting, it has also been identified as an angiogenetic switch molecule [32]. Synovial angiogenesis is thought to be a critical component in RA pathogenesis, contributing to pannus proliferation, infiltration of inflammatory leukocytes, as well as osteophyte formation [33]. In the present study, we demonstrate that cytoplasmic and extracellular HMGB1 appears early in the development of arthritis. We speculate that HMGB1 might be a major contributor to pannus formation in chronic arthritis.

Surprisingly, although our immunohistochemical analyses demonstrate the abundant presence of extranuclear HMGB1 throughout the inflamed synovium at a protein level, assessment with *in situ *hybridization reveals that the predominant upregulation of HMGB1 mRNA is restricted to synovial tissue adjacent to areas with cartilage and bone destruction. HMGB1 is an abundant nuclear protein. Most cells contain approximately 1 × 10^6 ^HMGB1 molecules in their nuclei [34], which may be translocated actively to the cytoplasm upon stimulation or passively by necrotic cells. *De novo *synthesis is thus not required for extranuclear expression. We speculate that the upregulated mRNA expression in the destructive zone may be of quantitative importance. It was recently demonstrated that osteoblasts themselves express HMGB1 as well as its signaling receptor RAGE (receptor for advanced glycation end products) and are capable of releasing HMGB1 [35]. Thus, HMGB1 represents a functional link between bone and immune cells. The precise role of HMGB1 in bone homeostasis and tissue destruction remains to be elucidated.

Similar to other cytokines, HMGB1 has differential tissue-specific activities. In addition to its potent pro-inflammatory capacities, HMGB1 has been accredited a role in tissue repair. As a signal of tissue damage, HMGB1 mediates tissue regeneration by inducing mesoangioblast migration and proliferation [36]. Synovial tissue has a strong capacity to regenerate, and not surprisingly mesenchymal stem cells have been isolated from synovium [37]. Recently, it was demonstrated that HMGB1 can induce myocardial regeneration after infarction; injection of HMGB1 into mouse hearts after ischemic damage resulted in the formation of new myocytes by inducing cardiac stem cell proliferation and differentiation [38]. Inflammation is the common driving force leading to cartilage, bone, and soft tissue destruction in chronic arthritis. Many factors involved in the regulation of normal tissue, in particular cartilage and bone, are dysregulated in arthritic diseases (reviewed in [39]). The persistence of synovial inflammation and its structural reorganization can be considered a remodeling process with abnormal tissue responses such as cartilage calcification and ankylosis that contribute to disease progression and loss of joint function. HMGB1 is a comprehensive cytokine that is able to orchestrate the regulation of both inflammation and tissue regeneration to promote wound healing, depending on different factors such as dose, spatio-temporal expression, target cells, and possibly even post-translational modifications of the secreted protein.

## Conclusion

We have demonstrated that extranuclear HMGB1 appears early in disease progression and is abundantly expressed in advanced arthritis. This suggests that, in chronic arthritis, HMGB1 may be considered an early mediator involved in both induction and perpetuation of inflammatory processes. The progressive destruction of cartilage and subchondral bone represents a major unsolved consequence of chronic arthritis. The marked presence of HMGB1 mRNA in the microenvironment of bone and cartilage destruction likely represents another functional link between inflammation and tissue destruction. Blockade of extracellular HMGB1 may offer a novel therapeutic alternative for the treatment of RA.

## Abbreviations

CIA = collagen-induced arthritis; HMGB1 = high-mobility group chromosomal box protein 1; LPS = lipopolysaccharide; Ig = immunoglobulin; IL-1β = interleukin-1-beta; PBS = phosphate-buffered saline; p.i. = post-immunization; RA = rheumatoid arthritis; TNF = tumor necrosis factor.

## Competing interests

The authors declare that they have no competing interests.

## Authors' contributions

KP helped conceive of the study, shared responsibility for study design coordination and was responsible for most of the experiments, data analysis and drafting of the manuscript. HEH helped conceive of the study and shared responsibility for study design coordination and drafting of the manuscript. ES assisted with the immunization, scoring, and collection of tissue specimens and contributed to the interpretation and discussion of data. RS assisted with the immunization, scoring, and collection of tissue specimens. MD assisted with and shared her knowledge of the *in situ *hybridization technique. A-CA assisted with the two-color staining. UA contributed to the interpretation and discussion of data. All authors read and approved of the final manuscript.
